# Sociodemographic and environmental health risk factor of COVID-19 in Jakarta, Indonesia: An ecological study

**DOI:** 10.1016/j.onehlt.2021.100303

**Published:** 2021-08-15

**Authors:** Zakianis, Fajriah Hanika Adzania, Sifa Fauzia, Gita Permata Aryati, Renti Mahkota

**Affiliations:** a Department of Environmental Health, Faculty of Public Health, Universitas Indonesia, Indonesia; b Department of Epidemiology, Faculty of Public Health, Universitas Indonesia, Indonesia

**Keywords:** COVID-19, Environmental health factor, Sanitation, Slum area, Sociodemographic factor

## Abstract

Since December 2019, the COVID-19 pandemic has rapidly emerged on a global scale. Many factors have influenced the spread of COVID-19. This research studies the sociodemographic and environmental health risk factors associated with COVID-19. The study used an ecological study design with subdistricts as its unit of analysis. The total population was 44 subdistricts. Data analysis used correlation and linear regression tests. The study results showed that the average COVID-19 incident rate in Jakarta is 99.8 per 10,000 population. Risk factors for the spread of COVID-19 were associated with population's high level of education (B = 3.094, *p* value<0.001), population density (B = 0.275, *p* value = 0.029), and slum area (B = 0.404, p value<0.001). The main risk factor for the spread of COVID-19 in Jakarta is high level of education, which can reflect a higher economic status to the population and a tendency to be more mobile. The government needs to enforce a mobility restriction to lessen the spread of COVID-19.

## 1 Background

The world is currently facing the COVID-19 pandemic. As per 21 June 2021, World Health Organization (WHO) reported 178,118,597 confirmed cases of COVID-19 worldwide, including 3,864,180 deaths [41]. In Indonesia, the number of COVID-19 patients also continues to rise. As per 21 June 2021, there have been 1,989,909 confirmed cases of COVID-19 with 54,662 deaths in Indonesia reported to WHO. In Jakarta alone, the number of cases has reached 479,043 with 7976 deaths [17].

COVID-19 is a disease caused by the coronavirus 2 (SARS-CoV-2) virus, a positive, single-stranded RNA virus that is not segmented. Coronavirus is included in the *Nidovirales* order, *Coronaviridae* family, *Orthocoronavirinae* sub-family, and is divided into groups α, β, γ, and δ in accordance with its serotypic and genomic characteristics. The virus is named after the wreath-shaped protrusion in the viral sheath [44].

COVID-19 transmission occurs in 2 ways: through droplets or close contact with an infected person. The droplets from the respiratory tract of an infected person can be transmitted to a healthy person when (s)he coughs or sneezes. Droplets can also stick to surfaces where the virus can live for roughly 3 h in droplet, 24 h on stainless steel, 2–3 days on cardboard, 3 days on plastic, and 4 days on copper [24,37]. The surrounding close environment of an infected person can serve as a source of transmission [40].

Some social health determinants can significantly affect the occurrence of COVID-19 for infected people and society because not everyone is affected by the COVID-19 pandemic [2]. These determinants can also affect pulmonary tuberculosis or other infectious diseases. Social determinants for pulmonary tuberculosis and COVID-19 can be related to housing and environmental conditions, physical environmental conditions, environment and safety, food security and malnutrition, costs and distance, social protection, culture and language, access to healthcare facilities, social isolation, and socioeconomic status [12].

### 1.1 Sociodemographic factors

Sociodemographic status is one of the risk factors affecting the increase of COVID-19 transmission. Sociodemographic factors include high population density, which increases human contact risk and make it difficult to maintain a minimum distance of 1–2 m [44], resulting in COVID-19 transmission [28], low education levels associated with positive COVID-19 test [9], and people with low socioeconomic status at risk of spreading COVID-19 [15]. The spread of COVID-19 is also strongly related to housing conditions [28]. One such condition is slum settlements, which are vulnerable to the spread of COVID-19 [6]. House occupancy density is also strongly related to COVID-19 and tuberculosis [12]. Previous studies related to infectious diseases through droplets, such as pulmonary tuberculosis, indicate that house occupancy density is one of the potential transmission risks of various diseases as it makes the transmission easier from one household member to another. Policies promoting affordable homes with standard sizes can help reduce the transmission of various diseases [7].

### 1.2 Environmental health vulnerability factor

Environmental health conditions could pose a risk to people's vulnerability in facing the COVID-19 pandemic. Environmental health factors include safe water quantity and quality, sanitation, and waste management as environmental health factors that affect COVID-19 transmission [49]. In addition to physical distancing and mask usage, handwashing with soap and running water is also a preventive measure at an individual level [36]. During the COVID-19 pandemic, adequate water supply is essential for ensuring the fulfillment of water demand for sanitation and handwashing purposes. Households that do not have access to safe water are one of the factors contributing to COVID-19 cases [38].

In the domestic waste management aspect, COVID-19 prevention measures performed at home resulted in the production of medical waste mixed with domestic waste. The use of a disposable mask can prevent droplets from being inhaled directly [44]. Alternatively, there is an increase in household medical waste production, especially masks and cleaning products bottle waste [28]. Quarantine policies in various countries resulted in shifting from conventional shopping to online shopping, which eventually increases packaging or inorganic waste at the household level [43]. In Indonesia, the imposed self-quarantine policy performed at home by patients with confirmed COVID-19 cases [45] potentially causes medical and domestic waste mixing. Waste produced by COVID-19 patients contains coronavirus, which can be the infection source [43]. In order to minimize exposure to potentially harmful or infectious waste, there needs to be an efficient waste management system at the household level. One aspect of good waste management to increase overall public health is the presence of good waste transportation by waste officers at the household level [5,14,34,39].

Good sanitation is vital to COVID-19 prevention. The presence of coronavirus in confirmed patient's feces indicates the possibility of fecal-oral transmission; thus, there need to be safety measures to avoid contact with patient feces [44]. Patient's feces, urine, and other waste need to be managed correctly by good sanitation facilities that ensure privacy and prioritize hygiene in their usage [[42], [46]]. Toilet usage and ending open defecation are important factors in maintaining proper sanitation [25].

Jakarta, which became one of the first area with positive COVID-19 cases in Indonesia, has also become the province with the highest number of positive cases. The number of cases is the accumulation of cases in Jakarta's subdistricts throughout 2020, where the lowest number of cases was found in the South Kepulauan Seribu subdistrict (53 cases), and the highest was in the Cengkareng subdistrict (4371 cases). With 44 districts and 267 subdistricts, the average confirmed cases per subdistrict in Jakarta were 2327 [17].

Multiple prior studies have observed relation between socialdemographic factors and COVID-19 spread rate in England, where low education and economic level were related to the spread rate [9]. Nonetheless, we believe that the result will be different from previous studies because Jakarta is a melting pot in Indonesia that has various culture and ethnicity which indirectly affects the sociodemographic aspects there. However, there is limited study regarding sociodemographic and environmental health risk factors of COVID-19, especially in Jakarta. Thus, this study further analyze the relationship between the sociodemographic and environmental health risk factors of the COVID-19 spread in Jakarta.

## 2 Method

This study was an ecological study with subdistricts as the unit of analysis. The dependent variable was the COVID-19 incidence rate per 10,000 residents in each subdistrict of Jakarta. Due to the limited data available, the independent variables were sociodemographic risk factors, including the proportions of different education levels, population density in each subdistrict, settlements on riverbanks, and families living in slums in each subdistrict. Environmental health risk factors measured in this study included the proportion of households using piped water sources, household waste transport by officers, households using latrines, and households that own infiltration holes as liquid waste disposal. The calculation of each variable and data source can be found in Table 1.

**Table 1:** Proportion Calculation per Variable.

Variables	Calculation	Data Source
COVID-19 incidence rate per 10,000 residents in each subdistrict	$\frac{\sum \mathrm{COVID}-19\;\mathrm{case}}{\sum \mathrm{population}\;\mathrm{per}\;\mathrm{subdistrict}}\;x\;10,000$	Jakarta Provincial Health Office https://riwayat-file-covid-19-dki-jakarta-jakartagis.hub.arcgis.com/
Primary education level proportion (do not go to school, have not gone to school, and primary level education)	$\frac{\begin{array}{c} \sum \mathrm{resident with primary education}\\ \mathrm{level} \end{array}}{\sum \mathrm{population}\;\mathrm{per}\;\mathrm{subdistrict}}$	Jakarta Provincial Office Integrated Data Portal Open Data https://data.jakarta.go.id/dataset/jumlah-penduduk-dki-jakarta-berdasarkan-pendidikan/resource/6e0301ac-4a66-4809-b6ee-b77c0e06d356
Secondary education level proportion (Junior High School and Senior High School)	$\frac{\begin{array}{c} \sum \mathrm{resident with secondary education}\;\\ \mathrm{level} \end{array}}{\sum \mathrm{population}\;\mathrm{per}\;\mathrm{subdistrict}}$	Jakarta Provincial Office Integrated Data Portal Open Data https://data.jakarta.go.id/dataset/jumlah-penduduk-dki-jakarta-berdasarkan-pendidikan/resource/6e0301ac-4a66-4809-b6ee-b77c0e06d356
Higher education level proportion	$\frac{\begin{array}{c} \sum \mathrm{resident with higher education}\;\\ \mathrm{level} \end{array}}{\sum \mathrm{population}\;\mathrm{per}\;\mathrm{subdistrict}}$	Jakarta Provincial Office Integrated Data Portal Open Data https://data.jakarta.go.id/dataset/jumlah-penduduk-dki-jakarta-berdasarkan-pendidikan/resource/6e0301ac-4a66-4809-b6ee-b77c0e06d356
Population density	$\frac{\sum \mathrm{population}\;\mathrm{per}\;\mathrm{subdistrict}\;}{a\mathrm{rea}\;\mathrm{per}\;\mathrm{subdistrict}}$	Jakarta Provincial Office Integrated Data Portal Open Data https://data.jakarta.go.id/dataset/datadkimenurutkepadatanpenduduk/resource/03266a68-1c1b-4848-aebe-f671e4e41ee6
Slum area proportion	$\frac{\begin{array}{c} \sum h\mathrm{ouseholds}\;\mathrm{living}\;\mathrm{in}\;\mathrm{slu}m\;area\;\\ \mathrm{per}\;\mathrm{subdistrict}\; \end{array}}{\sum h\mathrm{ouseholds}\;\mathrm{per}\;\mathrm{subdistrict}}$	Village Potential Data 2018
Riverbank settlement proportion	$\frac{\begin{array}{c} \sum households\;living\;in\;riverbank\;\\ \;settlements\;per\;subdistrict\; \end{array}}{\sum h\mathrm{ouseholds}\;\mathrm{per}\;\mathrm{subdistrict}}$	Village Potential Data 2018
Waste transport by officer proportion	$\frac{\begin{array}{c} \sum \mathrm{waste}\;\mathrm{transported}\;\mathrm{by}\;\mathrm{officer}\\ \;\mathrm{in}\;\mathrm{subdistricts}\;\mathrm{per}\;\mathrm{district}\; \end{array}}{\sum \mathrm{subdistricts}\;\mathrm{per}\;\mathrm{district}}$	Village Potential Data 2018
Latrine usage proportion	$\frac{\begin{array}{c} \sum \mathrm{subdistricts using latrine}\\ \mathrm{per}\;\mathrm{district}\; \end{array}}{\sum \mathrm{subdistricts}\;\mathrm{per}\;\mathrm{district}}$	Village Potential Data 2018
Availability of infiltration holes as liquid waste disposal proportion	$\frac{\begin{array}{c} \sum \mathrm{subdistrict having infiltration holes}\\ \;\mathrm{as}\;\mathrm{liquid waste disposal}\;\mathrm{per}\;\mathrm{district} \end{array}}{\sum \mathrm{subdistricts}\;\mathrm{per}\;\mathrm{district}}$	Village Potential Data 2018
Proportion of households with safe piped water for drinking water	$\frac{\begin{array}{c} \sum \mathrm{subdistricts using piped water}\\ \mathrm{for drinking water}\;\mathrm{per}\;\mathrm{district} \end{array}}{\sum \mathrm{subdistricts}\;\mathrm{per}\;\mathrm{district}}$	Village Potential Data 2018
Proportion of safe piped water for clean water source	$\frac{\begin{array}{c} \sum \mathrm{subdistricts using piped water}\;\\ \mathrm{for clean water souce}\;\mathrm{per}\;\mathrm{district} \end{array}}{\sum \mathrm{subdistricts}\;\mathrm{per}\;\mathrm{district}}$	Village Potential Data 2018

Assuming little to no change in the data, the latest data from the National Bureau of Statistics in 2018 were used as the independent variable. The use of village potential data was based on permission license number 54/LADU/0000/05/2019.

The population in this study consisted of 44 subdistricts spread across five municipalities and one district in Jakarta. The population included all administrative areas in Jakarta that have clear territorial boundaries, residents who live in the said area, and a legitimate and sovereign government [29].

Data were presented in tables based on distribution per variable for the dependent and independent variables. Data were analyzed using correlation and linear regression tests [19]. Two types of correlation test were used, Pearson, and Spearman correlation. The Pearson correlation test is used for variables with a normal distribution, while the Spearman correlation test is used for data that are not distributed normally. Multivariate analyze was performed using multiple linear regressions to determine sociodemographic and environmental health risk factors with a COVID-19 incidence rate in DKI Jakarta.

### 2.1 Ethical clearance

This study has been ethically approved by the Ethical Commission of Research and Public Health Service Faculty of Public Health Universitas Indonesia with permission number UN2·F10/PPM.00.02/202.

## 3 Results

The average number of confirmed positive cases per subdistrict was 2327 people, or 1% of the population. The incidence rate of COVID-19 per 10,000 people in Jakarta was higher compared to Indonesia's national average of only 21 per 10,000 people. The lowest incidence rate of COVID-19 in Jakarta was found in the North Kepulauan Seribu subdistrict (37.91 per 10,000 people), while the highest was found in the Cempaka Putih subdistrict (179.39 per 10,000 people) (Table 2).

**Table 2:** Descriptive Statistics of Sociodemographic and Environmental Health Factors in Jakarta, Indonesia, in 2021 (*n* = 44).

Variable	Mean	Median	SD	Min – Max
Sociodemographic factors				
Primary education level proportion	24%	23%	5%	17%–43%
Secondary education level proportion	59%	60%	4%	47%–67%
Higher education level proportion	17%	18%	6%	5%–36%
Population density (person/km^2^)	19,874	19,065	11,075	2894–60,624
Slum area proportion	6%	3%	9%	0%–42%
Riverbank settlement proportion	1%	0%	1.7%	0%–30%

Environmental health factors				
Proportion of piped water as a clean water source in households	40%	37%	40%	0%–100%
Household latrine usage proportion	100%	100%	0%	100%
Household septictank usage proportion	99%	100%	4%	80%–100%
Availability of infiltration holes as liquid waste disposal proportion	49%	36%	43%	0%–100%
Waste transport by officer proportion	100%	100%	0%	100%
Temporary waste shelter availability proportion	82%	100%	23%	33%–100%

**Graph 1:**
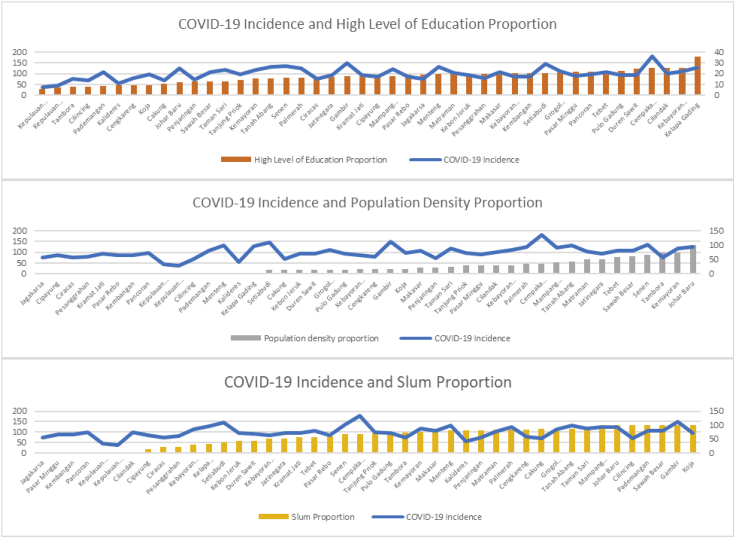
Sociodemographic Factors and COVID-19 Incidence.

**Graph 2:**
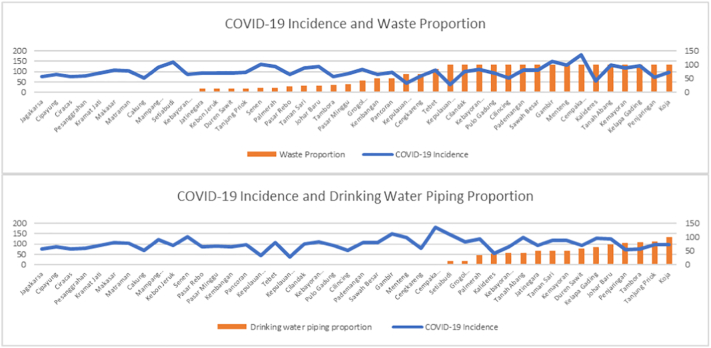
Environmental Health Factors and COVID-19 Incidence.

The average proportion of the population with secondary level education was 59%, higher than the national average of 48%. In addition, the average proportion of the population with higher education level was 17%, higher than the national average of 13% [31]. The average population density in Jakarta was very high, reaching 8500 persons/km^2^ [47] or more than 1000 persons/km^2^ [48], and higher than Indonesia's national average population density (140 people/km^2^) [32,33]. The average number of people living in slums in Jakarta was 6%, lower than the national average of 8% [30]. Meanwhile, the average population in Jakarta who lives near the riverbank area was 1% (Graph 1, Graph 2).

The average proportion of latrine usage and waste transport by officers showed an excellent result of 100% [39]. In Jakarta, the proportion of clean water piping was still categorized as poor, with only 40% coverage [18]. Similarly, results concerning the proportion of infiltration hole availability as household liquid waste disposal were merely 49% [23]. However, the proportion of septic tank usage in Jakarta showed good results, reaching an average of 99% [18].

Based on the data presented in Table 3, the correlation test result between the proportion of low education level, higher education level, very high population density, and slum areas with COVID-19 incidence rate had a significant value of <0.05, which shows a significant correlation. The strength of the relation could be seen from the correlation coefficient value. In the low education level proportion with COVID-19 incidence rate, the correlation coefficient was −0.627 (significant level < 0.001), showing that the relation between both data was 62.7% negatively related very significantly, meaning that the lower proportion of primary education level will result in a higher incidence rate of COVID-19.

**Table 3:** Relationship between COVID-19 Incidence Rate and Sociodemographic and Environmental Health Risk Factors in Jakarta, Year 2021 (*N* = 44).

Variable	COVID-19 Incidence Rate	
	Correlation Coefficient	p value
Primary education level proportion^a^	−0.627	0.000⁎
Secondary education level proportion^a^	−0.012	0.936
Higher education level proportion^a^	0.481	0.001⁎
Very high population density proportion^a^	0.377	0.012⁎
Riverbank settlement proportion^a^	−0.162	0.293
Slum area proportion^a^	0.355	0.018⁎
Waste transport by officer proportion	0	0
Latrine usage proportion	0	0
Availability of infiltration holes as liquid waste disposal proportion^b^	0.176	0.252
Proportion of households with safe piped water for drinking water^b^	0.167	0.277
Proportion of safe piped water for clean water source ^b^	0.207	0.179

⁎ *p ≤ 0.05.*

a Pearson correlation.

b Spearman correlation.

Linear regression result showed that the independent variables that entered the final regression model were the proportion of low education level, higher education level, high population density, and slum area (Table 4). The determinant coefficient (*R square*d) showed a value of 0.568, meaning that the regression model can explain that having higher education level and living in slum area with high population density increase 56.8% of COVID-19 incidence rate (Table 4). Meanwhile, higher education level alone can increase the incidence rate of COVID-19 to 68%.

**Table 4:** Final Model of the Relationship between COVID-19 Incidence Rate and Sociodemographic and Environmental Health Risk Factors in Jakarta, Year 2021.

Model	Unstandardized Coefficients		Standardized Coefficients B	t	Sig	95% CI	Adjusted R^2^
	B	Standard error	Std. Error Beta				
Proportion of higher education level	3.094	0.488	0.681	6.345	<0.001	2.108–4.079	0.568
Proportion of high population density	0.275	0.122	0.246	2.259	0.029	0.029–0.522	
Proportion of slum area	0.404	0.093	0.501	4.321	<0.001	0.125–0.593	
Constant	17.412	11.440		1.522	0.136	5.710–40.533	

Dependent Variable: COVID-19 incidence rate per 10,000 population.

## 4 Discussion

The study results showed that higher level of education is related to the occurrence of COVID-19. The results showed a different outcome from a study conducted in England, where a low education level was related to COVID-19-positive test results [9]. One of the indicators of higher socioeconomic status was residents who can work and travel [16]. People with higher education levels tend to have office-based jobs and work in office areas. Jakarta has imposed Large-Scale Social Restrictions since March 2020; however, since the 5^th^ June 2020, workers had been able to normally operate in their offices with a maximum capacity of 50% [21]. In July 2020, 68 reported cases of COVID-19 occurred due to office clustering, with 440 workers diagnosed as COVID-19 positive [11]. In addition, because people who have a higher level of education generally have a better socioeconomic status, they generally tend to have high mobility [1,27], which increased the risk of COVID-19 infection [4,26]. Accordingly, people traveling outside home could increase the risk of COVID-19 transmission and spread [8].

The level of population density was related to COVID-19 occurrence. The result was in line with previous study conducted in Algeria [20] which showed strong correlation between population density and the number of positive COVID-19 cases. One of the preventive measures against COVID-19 infection involved maintaining a physical distance of at least 1.6–2 m [8]. High population density made physical distancing difficult, thus increasing the transmission rate of COVID-19.

A relationship also found between slum areas and COVID-19 cases. Slums were characterized by residential areas that do not have adequate access to safe water, improper sanitation, poor housing quality, high population density, and illegal housing status [35]. Settlements with high population density and lack of adequate sanitation could increase the risk of COVID-19 transmission [3]. Residential settlement conditions were also one of the socioeconomic determinants. Previous studies in the United States stated that groups with low socioeconomic had a higher risk of being infected with COVID-19 because the group was affected by comorbid diseases that could worsen the condition. In times of communicable disease pandemics such as COVID-19, distance restrictions should be applied on a large scale worldwide. However, this was unlikely in slums due to the high density of buildings in the area [13].

The correlation test result between the proportion of households with piped water as a source of drinking water and clean water showed no significance with COVID-19 infection. This could happen because COVID-19 was being transmitted through droplets instead of water [40]. In COVID-19 prevention, water is needed to wash hands. However, people can use hand sanitizer if water was not available for washing hands [36]. The consistency and accessibility of Jakarta's piped water supply are both low. In Jakarta, less than a third of the population has access to piped water in their homes [10]. Based on statistics by Statistics Indonesia, besides metered pipe water (PAM/PDAM) people in Jakarta got clean water from non-metered piping (2%) and ground water using pumps (58%) [29].

Risk factors related to the spread of COVID-19 were the level of higher education, population density, and slum area. This was supported by a previous study conducted in Kenya, which showed that slum dwellers had worse health and socioeconomic conditions than other social groups due to limited access to education, employment, water, and sanitation [22]. Therefore, efforts were needed to prevent the spread of COVID-19 by ensuring the availability of safe water, good waste management, and the need to improve hygiene in slums [42].

The use of correlation study design limited the study to only utilize aggregate data; thus, the data obtained only applies to a certain population and was unable to represent individual exposure specifically. Moreover, this study's correlation study design could only examine the initial relationship between independent and dependent variables. The results shown in this study only described the initial severity of the studied variables. Therefore, further research is needed on the causal relationship between independent and dependent variables at an individual level, for example, using a cross-sectional, case-control, or cohort study. Independent variables should be added in accordance with previous research, such as household income levels, food safety and malnutrition, social protection, cost and distance, access to healthcare facilities, and social isolation [12].

## 5 Conclusion

Based on this study, factors related to COVID-19 cases in Jakarta were higher education level, population density, and slums. There needs to be a unique approach for residents with higher education levels, especially those with high office activity and mobility levels. Strict regulations on mobility restrictions need to be enforced. Governments in areas with high-level population density must increase public awareness to avoid crowds and maintain personal and environmental hygiene. These local governments should also forbid self-isolation at home for those with non-symptomatic COVID-19. Instead, they need to provide designated isolation locations. In the future, the government is encouraged to organize slums, especially regarding environmental health, such as providing safe water and efficient solid and liquid waste management. In addition, local governments are expected to provide a decent and affordable housing for the poor.

## Declaration of Competing Interest

The authors declare that they have no known competing financial interests or personal relationships that could have appeared to influence the work reported in this paper.
